# SNPs for breast cancer risk assessment

**DOI:** 10.18632/oncotarget.22278

**Published:** 2017-11-03

**Authors:** Jack Cuzick, Adam Brentnall, Mitchell Dowsett

**Affiliations:** Jack Cuzick: Centre for Cancer Prevention, Wolfson Institute of Preventive Medicine, Queen Mary University of London, London, UK

**Keywords:** breast cancer risk, common low penetrance genetic polymorphisms, SNP panels, SNP calibration, risk adapted screening

Accurate risk assessment for breast cancer is based on information from three domains. The first consists of classical factors which can be determined from interview or questionnaire, and include age, family history of breast or ovarian cancer (with age of onset), menopausal status, body mass index, use of hormone replacement therapy, age of first childbirth and prior proliferative benign breast disease (with or without atypia). These factors can be combined in programmes such as the Tyrer-Cuzick (TC) model, which is freely downloadable ([1] and www.ems-trials.org/riskevaluator/). The second is mammographic density, originally developed by Wolfe but now available in both quantitative area based (Cumulus) and volumetric forms (Volpara). The third is a panel of common low penetrance single nucleotide polymorphisms (SNPs). Over 100 of these have now been validated [2,3] and they play a qualitatively very different role than the very rare mutants such as *BRCA1* or *BRCA2* and the dozen or so immediate risk genes such as *ATM*, *CHEK2* and *PALB2* [4], in that they are common and individually most carry a minimal added risk of the order of 5 to 10%. However, in combination these common variants provide useful risk information of the same magnitude as classical factors or breast density.

Several studies have indicated that these three domains are largely independent [5], so much can be gained by using them together. Commercially available tests for a selected SNPs panel are not yet generally available, but they can mostly be selected from the OncoArray, which contains more than half a million SNPs [6]. We have used 88 of these that have been validated in large studies to create our SNP score by multiplying the risk from each of these alleles (which can be above or below unity). The occurrence of different SNPs is largely independent, but much less is known as to whether their effects are independent or whether interactions between the risks for different SNPs exist, and more work is needed to determine this. Linking specific SNPs to different types of breast cancer (eg based on ER or HER2 status) is also an important goal.

The present study looked at women at high risk who participated in one of two breast cancer prevention trials using tamoxifen. Risk from classical factors was determined using the TC model and combined with a SNP score based on the OncoArray assessments, but data on mammographic density was not available. The results indicated that the SNP score provided useful additional information not contained in the TC model, but the overall prediction was somewhat optimistic and calibration was poor. This was also seen for another polygenic risk score in a case-control study of women from two other prevention trials [7]. However, other studies have not seen a loss in calibration (8, van Veen et al submitted). For example, in a case-control study from a UK family history clinic [8], a score which used only 18 SNPs was found to also have good predictive value independent TC variables, and the results were well calibrated. Only 25% of the observed information in the 88 SNP score we used above was captured in these 18 SNPs, indicating the value of larger panels. However it is less clear how much more can be gained by further extension, as the new SNPs will have less predictive value. As the SNP score appears to be independent of other factors, scores based on other SNPs should also be valid and version 8 of the TC model allows the introduction of a SNP score-derived relative risk based on any panel of individual genes.

There was some evidence that SNP88 was more predictive for ER positive tumours and for women not taking tamoxifen, but neither of these interactions was significant. A major remaining challenge is to find SNP scores that predict different types of breast cancer and differential response to different preventive agents (eg tamoxifen vs aromatase inhibitors).

Accurate risk prediction is essential for risk adapted screening algorithms, which are currently being explored in different settings. In the long-term, breast screening programs should be expanded to provide risk assessment and take on a breast cancer prevention activity. This is would be best based on a single risk assessment at the time of the first mammogram around age 40 to 50y, and would include classical risk factors as provided in the TC model, as well as density based on the first mammogram and a SNP score. In addition to identifying high-risk women who might benefit from preventive therapy, this approach could be used to guide the frequency of subsequent screening and determine which women need additional types of screening such as MRI, and which may need little or no screening at all.
